# Some Medical Aspects of the Peace Conference

**Published:** 1907-10-26

**Authors:** 


### Some Medical Aspects of the Peace Conference.

As medical men we have, perhaps, little to do with the purely theoretical side of the Peace Conference. As lovers of peace, as humanitarians in the best sense of the term, we may welcome the encouraging signs of a desire on the part of the great nations for a settlement of disputes by methods other than the rude arbitrament of the torpedo or the quick-firing gun. But there are questions which were broached at the Conference which affect us more closely. They are points upon which the medical man may venture to express an opinion with a greater degree of confidence, perhaps also with a better right to a hearing, than the mere layman.

Baron Larrey, the prince of Red Cross surgeons, to whom humanity owes more than to the great master whose tools he had to repair, touched the heart of the question of humanitarian work on the battlefield when he declined to certify as fit for service the men whose lives he had saved by boiling down the chargers of Napoleon's hussars into soup. The soldier looks to the surgeon to aid him in balancing accounts after a battle. On the one hand we are making war more sanguinary than it has ever before been, on the other we are attempting to rid the battlefield of its gruesomeness by helping and succouring the wounded. The medical staff of an army stands neutral between the combatants: its help is as much for the enemy as for its own side. If we adopt the principle that in modern warfare the endeavour is to incapacitate, not necessarily to kill, the opposing force, the medical staff should be placed on a much more neutral footing than it is at present. The wounded man who has been repaired in the ambulance tent is no longer on the same standing as the non-wounded combatant. The very fact that he has had to seek succour from the neutral body attached to the force should render the former a non-combatant, holding the same privileges and having the same obligations as any other civilian. It is a travesty of humanitarian sentiment to maintain that the surgeon is there to repair the tool, to make it once more fit to become food for the cannon, and it will be an advance towards the highest ideal when a rule is laid down to the effect that those who have been succoured by the Red Cross are *ipso facto* incapacitated from further service so long as the campaign is in progress.

Equally important is the question of safeguarding the rights and privileges of the Red Cross itself. In every war abuse of the neutrality of the ambulance service has been conspicuous, and unless definite international action is taken in the matter, such abuses will continue to be prevalent in every future war. It is idle to assert that scrupulous consideration of the Geneva Convention can never be insisted upon. It must be insisted upon, and all infringements, not merely of the letter but equally of the spirit of that Convention must be summarily dealt with. There are, no doubt, grave difficulties in connection with this matter. A base hospital, for instance, can never be entirely separated from the army commissariat, and the question of supplies is one of the most difficult to deal with. At present the train which brings hospital supplies carries also munitions of war, and the rations which are issued to the ambulances come from the quartermaster's stores. The wrecking of a supply train, therefore, may mean a serious difficulty to the Red Cross department. Some limitation should be agreed upon. Either the ambulance should have its own special commissariat department, should get its stores despatched in special trains, flying its own flag and exempt from attack, and should have its own special base, or it should, in some other way, be distinctly dissociated from the essentially hostile, military element.

These are questions which are more efficiently discussed at a Peace Conference, perhaps, than at an international Red Cross Conference, where individual, private zeal is more conspicuous than official support and international ratification. At present we, as medical men attached to a field force, have obligations which are loyally adhered to. We have privileges which are often impossible and usually difficult to enforce. We cannot expect to be permitted a say in military matters, but we can at least claim to have a voice in the safeguarding of the rights of those whom we have to treat, and as humanitarians to do our utmost to mitigate the terrible results of national calamities which we are powerless to prevent.

